# Preparation and Preliminary Application of Epitope Peptide-Based Antibody against *Toxoplasma gondii* GRA3

**DOI:** 10.3390/tropicalmed8030143

**Published:** 2023-02-27

**Authors:** Ru Wang, Minmin Wu, Haijian Cai, Ran An, Ying Chen, Jie Wang, Nan Zhou, Jian Du

**Affiliations:** 1 Department of Biochemistry and Molecular Biology, School of Basic Medical Sciences, Anhui Medical University, Hefei 230032, China; 2 Research Center for Infectious Diseases, School of Basic Medical Sciences, Anhui Medical University, Hefei 230032, China; 3 Provincial Key Laboratory of Zoonoses of High Institutions in Anhui, Anhui Medical University, Hefei 230032, China; 4 Center for Scientific Research of Anhui Medical University, Anhui Medical University, Hefei 230032, China

**Keywords:** *Toxoplasma gondii*, GRA3, epitope peptide antibody, antigenic epitope, Toxoplasmosis

## Abstract

*Toxoplasma gondii* dense granule protein GRA3 has been shown to promote *Toxoplasma gondii* transmission and proliferation by interacting with the host cell endoplasmic reticulum (ER) through calcium-regulated cyclophilin ligands (CAMLG). Although many studies have focused on the interaction between the host cell endoplasmic reticulum and GRA3, no polyclonal antibodies (PcAbs) against GRA3 have been reported to date. According to the antigenicity prediction and exposure site analysis, three antigen peptide sequences were selected to prepare polyclonal antibodies targeting GRA3. Peptide scans revealed that the major antigenic epitope sequences were ^125^ELYDRTDRPGLK^136^, ^202^FFRRRPKDGGAG^213^, and ^68^NEAGESYSSATSG^80^, respectively. The GRA3 PcAb specifically recognized the GRA3 of *T. gondii* type Ⅱ ME49. The development of PcAbs against GRA3 is expected to elucidate the molecular mechanisms by which GRA3 regulates host cell function and contribute to the development of diagnostic and therapeutic strategies for toxoplasmosis.

## 1. Introduction

*Toxoplasma gondii* (*T. gondii*) is a widespread intracellular parasite that infects animals, including humans. Most infections caused by *T. gondii* are usually asymptomatic; in immunocompetent hosts, it tends to cause a self-limiting disease [1]. *T. gondii* can establish chronic infection in organs such as the brain or heart during the infection of host cells [2]. Central nervous system infection with *T. gondii* can lead to toxoplasmic encephalitis. Infection with *T. gondii* causes endoplasmic reticulum (ER) stress and the activation of the unfolded protein response (UPR), ultimately leading to apoptosis of the host cell [3,4]. The dense granule protein GRA3 is one of the secreted proteins that promote *Toxoplasma* virulence. GRA3 has been found to interact with the endoplasmic reticulum (ER) of host cells through calcium-regulated procyclin ligands (CAMLG) [5,6]. GRA3 is a 29 kDa dense granule protein localized to parasitic vesicle membranes and the intravacuolar network. It not only aids in nutrient uptake in host cells, but has also been shown to exert virulence in type II strains [5,7]. *T. gondii* has three organelles that release proteins in sequence during the invasion and development of the PV. These organelles are the micronemes (which release proteins for initial recognition and attachment), rhoptries (which release proteins during the initial PV formation), and dense granules (which are released after the PV formation). Dense granules are called dense granules because they are very dense when viewed with transmission electron microscopy (TEM) [8,9,10,11]. GRA3 plays an important role in *T. gondii* after the invasion of host cells and has high transcript levels in *T. gondii*. GRA3 is mainly expressed in the tachyzoite phase and may be a prerequisite for *T. gondii* replication [12,13]. GRAs resist host cell attacks against *T. gondii*, for example, by resisting the acidification and lysis of host cell lysosomes, thus ensuring that *T. gondii* can survive within the cell. Since GRAs have good immunogenicity in the host, they are an important part of the antigens that make up the blood circulation after host infection [14]. GRA proteins are known to powerfully stimulate the immune response of the host. These molecules are powerful antigens that trigger strong B- and T-cell responses in the presence of infection [15,16]. Protein antigens contain epitope structures used to mediate the immune response, including those used by T, B, and NK cells, and cytotoxic T lymphocytes (CTL), and may also contain structures that are detrimental to protective immunity. Thus, the study of antigenic epitopes in *T. gondii* has not only improved our understanding of the reaction between antigens and antibodies, the function and structure of antigens, and many other aspects of immunology, but has also contributed to the development of new vaccines and diagnostic reagents [17]. Polyclonal antibodies (PcAbs) against GRA3 are not yet commercially available, which makes it difficult to understand the molecular mechanisms governing the function of GRA3. This lack of understanding of GRA3 function also affects the development of possible clinical applications, such as diagnostic kits. In this study, we characterized and created the immunochemical properties of antibodies targeting GRA3. We predicted and analyzed the GRA3 linear antigenic epitope by bioinformatics software, synthesized the epitope peptide by peptide solid phase synthesis, and the obtained epitope peptide was conjugated to the carrier protein bovine serum albumin (BSA), thereby immunizing New Zealand rabbits to prepare GRA3 polyclonal antibodies, which were applied for Western blotting, immunofluorescence detection, and immunoprecipitation assay.

## 2. Materials and Methods

### 2.1. Parasites 

*Toxoplasma gondii* ME49 tachyzoites were cultivated in human foreskin fibroblast (HFF) cells in Dulbecco’s modified Eagle’s medium (DMEM) which contained 1% penicillin–streptomycin amphotericin B (Biological Industries, Beit Haemek, Israel) and 10% fetal bovine serum (FBS). N2a and HEK293T cells were cultivated and held in DMEM which included 1% penicillin–streptomycin–amphotericin B (Biological Industries, Beit Haemek, Israel) and 10% FBS at 37 °C in a 5% CO_2_ humidified atmosphere. All cell culture processes are ensured to be free of *Mycoplasma* contamination and that cells are in good condition, thus preventing contamination from affecting experimental results. 

### 2.2. Plasmids and Protein Expression

The open reading frame (ORF) of *T. gondii* GRA3 (GenBank^TM^ ID XP_002366371.1) was amplified by RT-PCR from the total RNA of *T. gondii* (ME49 strain). The ME49 strain was first blown off the Vero cells and broken up by blowing with a 1 mL syringe; the cells were separated from the ME49 strain by centrifugation at 800 rpm/min for 3 min; the supernatant obtained was centrifuged at 3000 rpm/min for 5 min; and 500 μL of Trizol was added to the obtained precipitate, followed by 200 μL of chloroform. The supernatant was transferred to a new EP tube, 500 μL of isopropanol was added and left at room temperature for 10 min, followed by centrifugation at 12,000 rpm for 15 min, and the supernatant was discarded. To the white flocculent precipitate obtained after centrifugation, 1 mL of ethanol and DEPC water (750 μL of ethanol and 250 μL of DEPC water) was added and washed with shaking. After washing and then centrifugation at 7500 rpm/min at 4 °C for 5 min, the supernatant was subsequently discarded and left open to air-dry for several minutes, and finally 20 μL of DEPC water at 55 °C to 60 °C was added to promote solubilization to obtain RNA of ME49 strain. The obtained RNA was then reverse-transcribed to obtain cDNA, and the upstream and downstream primers of GRA3 were designed using Primer Premier software, and the obtained cDNA was subsequently subjected to PCR reactions with the designed primers and Taq polymerase to obtain the GRA3 plasmid. The GRA3 plasmid obtained by PCR and the pGEX-6P-(27-4597-01) empty vector plasmid were double-cleaved using *XcoR*1 (Takara, Dalian, China) and *EcoR*1 (Takara, Dalian, China) endonucleases, and the cleaved fragments were added to DNA loading buffer for DNA agarose gel electrophoresis and observed under UV developer, and the target bands were cut off for gum recovery. The DNA fragments were ligated to the vector DNA fragments using T4DNA ligase to obtain the GRA3-GST plasmid. In the same way, the gene sequence encoding GRA3 was inserted into the prokaryotic expression vector pEGFP-C2 (BD Biosciences) and the pCMV-3Tag-2A vector (BD Bioscience), thus preparing for later antibody-specific detection and indirect immunofluorescence detection. The resulting plasmids was then transformed into *Escherichia coli* TOP10 (Invitrogen Corp, USA) and the monoclonal bacteria were selected and transferred to LB (Luria–Bertani) liquid medium containing ampicillin (50 mg/mL) in LB liquid medium with ampicillin resistance to screen for *E. coli* containing the target plasmids.

In order to obtain the recombinant protein, the pGEX-6p-1-GRA3 plasmid was transformed into Rosetta (DE3) host bacterial cells (Transgen, Beijing, China) and the monoclonal bacteria were selected and transferred to LB liquid medium containing ampicillin (50 mg/mL) in LB liquid medium with ampicillin resistance to screen for *E. coli* containing the target plasmids. The cultures were incubated overnight at 37 °C on a shaker. The screened *E. coli* were transferred to 200 mL LB liquid medium at 1:100 and incubated at 220 rpm at 37 °C. When the DO 600 nm value was between 0.4 and 0.6, IPTG was added at a final concentration of 0.1 mmol/L. A blank control was set up and the culture was induced at 30 °C for 8 h at 220 rpm. The bacteria cells were then centrifuged at 8000 rpm for 5 min at 4 °C, which allowed obtaining the bacteria cells. The resulting pellet was resuspended in lysis buffer (PBS in 1% Triton X-100) and the bacterial suspension was sonicated on ice at 400 W/s for a total sonication time of 10 min with 6 s intervals. Next, the lysates were centrifuged at 12,000 rpm for 20 min to separate the supernatant from the bacterial debris. The protein expression levels were analyzed by 15% SDS-PAGE electrophoresis and Coomassie Brilliant Blue R-250 staining. The results showed that in the inclusion bodies, the bacterial debris precipitated from the previous step was first resuspended with PBS and centrifuged twice, after which 5 mL containing 7 mL guanidine hydrochloride was added and shaken overnight at low speed, after which the solution from the previous step was added to a 4 M urea dialysis bag for dialysis reversion (the bag was preboiled in boiling water for 10 min and then washed with pure water), and then the bag was left in 4 M urea dialysis solution overnight at 4°C and then placed in 200 mL 3 M urea dialysate for 6 h at 4 °C, followed by 2 M urea dialysate for 4°C overnight, followed by 200 mL Tris solution for 6 h at 4 °C (repeated twice), followed by centrifugation of the fluid at 12,000 rpm for 15 min to separate the protein supernatant. The expression levels of the obtained protein supernatants were analyzed by 15% SDS-PAGE electrophoresis and Coomassie Brilliant Blue R-250 staining. By analysis, it was concluded that the target protein was present in the protein supernatant, and the supernatant was stored at 4 °C for subsequent experiments.

### 2.3. Main Reagents 

The main reagents are PVDF membrane (GE Healthcare, Solingen, Germany), HRP-conjugated secondary antibodies (Proteintech, Wuhan, China, 1:10,000), ECL blot detection system (Bio-Rad, Hercules, CA, USA), and Glutathione-Sepharose 4B beads (GE Healthcare, Solingen, Germany).

### 2.4. Rabbit Immunization and Polyclonal Antibody Production

Before antigen injection, rabbit blood was drawn through the ear marginal vein, and the serum was separated by centrifugation and stored at −80 °C. We dissolve and dilute 1 mg of three prepared peptide-BSA (bovine serum albumin) coupling with 1 mL of sterile saline, respectively, then mix it completely with 1 mL of Freund’s Complete Adjuvant (FCA) to form a stable emulsion, and inject it subcutaneously into the back of the neck of New Zealand rabbits at multiple points. Every week after the end of the initial immunization, mix the same 1 mL of peptide solution with 1 mL of FIA (Freund’s Incomplete Adjuvant) to emulsify, and then inject 1 mg of antigen into each rabbit by the same method. After the end of the third immunization, 500 μg of the three peptides were dissolved and diluted with 1 mL of sterile saline, respectively, and injected directly into the vein at the ear margin to strengthen the immunization, and four days later, serum was collected from the marginal vein of the rabbit ear and stored at −80 °C for the next experiments.

### 2.5. Determination of Antibody Potency

The three peptides of GRA3 and the whole protein solution after denaturation of inclusion bodies were diluted to 5 μg/mL with the coating solution; 100 μL per well was added dropwise to a 96-well plate and incubated overnight at 4 °C in a wet box. After washing the plate with phosphate buffer solution (PBST) containing 0.05% Tween-20 3 times (5 min/time), 200 μL of 1% BSA was added to each well. After washing as above, the prepared GRA3 peptides immune serum was diluted from 1:1000 to 1:64,000. The prepared rabbit pre-immune serum was also used as a negative control and diluted from 1:1000 to 1:64,000, with phosphate buffer saline (PBS) at 2-fold multiplicity, and negative and blank controls were set, incubated for 90 min at 37 °C and washed as above, and goat anti-rabbit-HRP IgG was diluted to 1:10,000. After washing as above, 100 μL was added to every well and kept hatched at 37 °C for 60 min. After washing as above, 100 μL of the substrate was added to each well and placed in a dark place for 5 min and then the termination solution was added at 50 μL/well, and the OD value was read at wavelength 450 nm with an enzyme marker within 5 min.

### 2.6. Antibody Specificity Assay

Total protein was extracted from N2a cells infected with ME49 tachyzoites or from N2a cells transfected with GRA3-GFP plasmid, respectively, and the proteins were separated by SDS-PAGE gel electrophoresis, and then transferred to PVDF membranes (GE Healthcare), and the membrane was washed 5 times (5 min/time) with 5% skimmed milk for 1 h TBST before using the prepared GRA3 polyclonal antibody (1:5000), and rabbit serum before immunization was used as a negative control, incubated overnight at 4 °C, washed as above, and then the membrane was transferred to goat anti-rabbit secondary antibody (Proteintech, Wuhan, China, 1:10,000) diluted 10,000 times and incubated for 2 h at room temperature, washed as above, and then developed by ECL blot detection system (Bio-Rad, Hercules, CA, USA), and the results were analyzed.

### 2.7. Application in Indirect Immunofluorescence Assay

After digestion and resuspension of Vero cells, we add 2.5 × 10^5^ cells/well to a 24-well plate with sterile coverslips, wait until the cells grow to about 70% of the plate, add ME49 tachyzoite suspension (about 1 × 10^5^/well), continue to incubate for 24 h, then wash 3 times with PBS (5 min/time), and add 200 μL 4% paraformaldehyde to each well. After washing, 200 μL of 0.2% Triton X-100 (Sigma, St. Louis, MO, USA) was added to each well for 20 min. After washing, 200 μL of 10% goat serum was added to every well and kept incubated for 2 h at 37 °C. After washing, 200 μL of the GRA3 PcAb 1:500) and Rhodamine-conjugated anti-SAG1 antibody (GTX38936; GeneTex, California, USA) were added to every well and kept incubated overnight at 4 °C. After washing, we add 200 μL of rhodamine red-labeled goat anti-rabbit secondary antibody (1:100) to each well, and incubate for 1 h at 37 °C avoiding light. After washing as above, we add 100 μL DAPI to each well to stain the nuclei for 5 min. After washing as above, we remove the coverslip, add the appropriate amount of anti-fluorescence quencher dropwise to seal the slide, and observe under a fluorescence microscope.

### 2.8. Immunoprecipitation Assay 

HEK293T cells co-transfected with the MYC and GRA3-MYC plasmids were lysed in lysis buffer (150 mM NaCl, 50 mM HEPES, pH 7.4, 2 mM EGTA, and 1% Triton X-100) including Complete™ protease inhibitor (Roche Applied Science, Indianapolis, IN, USA). The supernatant was collected and the obtained supernatant was ligated with the corresponding antibody binding protein A/G plus agarose (Sparkjade, Shandong, China) for 4 h at 4 °C. The immunoprecipitates were then washed three times with pre-chilled 0.1% Triton X-100 lysis buffer and three more times with PBS. Western blot analysis was performed on the bound proteins.

### 2.9. Statistical Analysis

The amino acid sequence of *Toxoplasma gondii* GRA3 protein was obtained from the NCBI database (GenBank: XP_002366371.1), and the DNAstar software(Madison, WI, USA) was used to predict the antigenic epitopes of GRA3 amino acids based on their physicochemical properties such as hydrophilicity; ELYDRTDRPGLK-C (125–136), FFRRRRPKDGGAG-C (202–213), and NEAGESYSSATSG-C (68–80) were finally identified. Experimental data from antibody potency assays were analyzed using GraphPad Prism 5.0 software (San Diego, CA, USA), and all data results were obtained from three replicate experiments.

## 3. Results 

### 3.1. Peptide Design and Synthesis 

**Figure 1:**
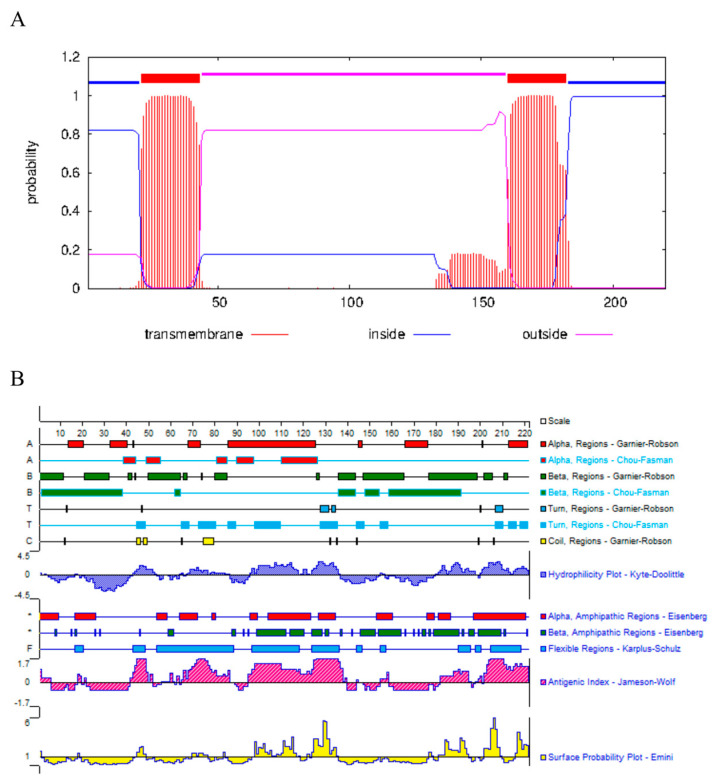
Sequence analysis of GRA3. (**A**) Transmembrane domain analysis. Two transmembrane domains are shown. (**B**) Antigenicity analysis.

In general, the antigenicity of peptides is closely related to the physicochemical properties and structure of the amino acid sequence, and the hydrophilic region on the protein surface is the only structure that can interact with antibodies, and the affinity of antibody recognition is proportional to the continuity of the amino acid residues that make up the protein [18,19]. Three dominant antigenic determinant cluster peptides with good hydrophilicity and strong antigenicity were used for the preparation of peptide antibodies. Then, we synthesize the peptides by peptide solid-phase synthesis (Figure 1A,B). The peptides were subsequently used for rabbit immunization and polyclonal antibody production (Figure S1).

### 3.2. Plasmid Construction and Determination of Antibody Potency

**Figure 2:**
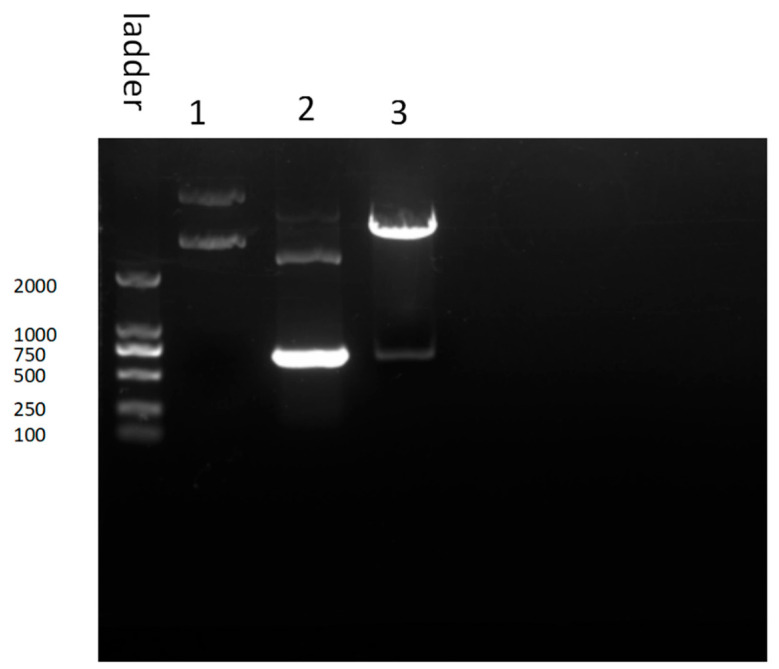
Construction of GRA3-GST plasmid. Line 1 reflects the agarose gel electrophoresis results of GST-GRA3 plasmid mixed with 10 × Loading Buffer. Line 2 reflects that after PCR of the GRA3-GST plasmid, the band of GRA3 was 680 bp. Line 3 reflects the results of agarose-gel electrophoresis of GRA3-GST plasmid digested by *Xco*R1 and *Eco*R1 enzymes and mixed with 10× Loading Buffer. The results were consistent with those of the PCR.

The results of PCR and enzymatic agarose gel electrophoresis showed that GRA3 was about 680 bp (Figure 2). In addition, the DNA sequencing also confirmed that the GRA3-GST plasmid was successfully constructed. *E. coli* prokaryotic expression systems, due to their simplicity, high expression capacity, and low cost, are currently the most widely used protein expression methods. The plasmid was then transformed into *Rosetta* (DE3) host bacterial cells to express the recombinant protein. Bioinformatics analysis predicted that GRA3 is a hydrophilic protein with two transmembrane domains, but the GRA3 protein was mainly present in the precipitate as inclusion bodies in this experiment, probably due to the high prokaryotic expression, as it was too late to fold the protein structure correctly. The codon has been optimized for the subsequent experiments. However, optimization is difficult to improve solubility, and the protein became soluble after the removal of urea during the complex denaturation process, and numerous studies have shown that 30% of the folded protein was correct after the complexation. The soluble expression of the protein can be considered later by replacing it with a eukaryotic expression system depending on the need of the experiment.

### 3.3. Determination of Antibody Potency

**Figure 3:**
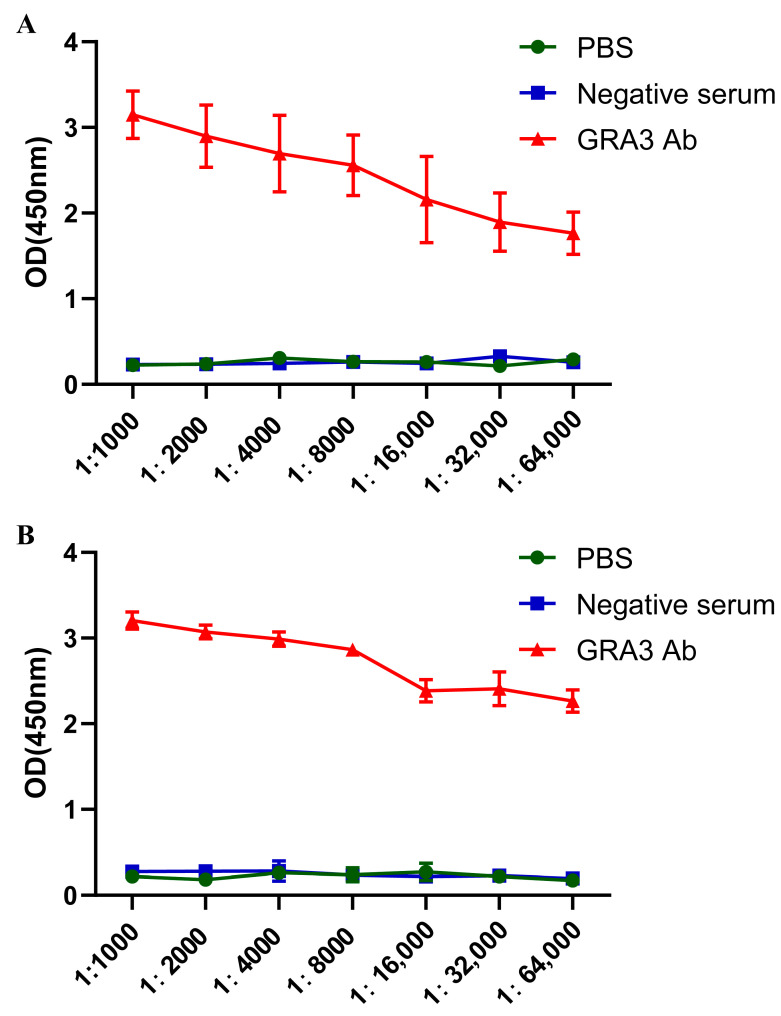
Determination of antibody potency. (**A**) The whole protein solution after denaturation of inclusion bodies were diluted to 5 μg/mL with the coating solution. The potency of antibodies in immunized rabbit sera was measured by indirect ELISA, and the dilution ratio of antibodies was adjusted to 1:64,000. Sera from normal rabbits and PBS were used as negative controls. (**B**) The three peptides of GRA3 were diluted to 5 μg/mL with the coating solution. The potency of the antibody in the immunized rabbit serum was detected by indirect ELISA at a dilution of 1:64,000. The serum of normal rabbit and PBS acted as negative control. The final result was consistent with group A.

The three peptides and the whole protein solutions of GRA3 were used for ELISA assays. The inclusion body protein and the three peptides were diluted to 5 μg/mL with the coating solution, and 100 μL of the coating solution containing the inclusion body protein or the three peptides was added to the empty ELISA plate, resulting in antibody potency determination. The OD450 results obtained from the ELISA assay of GRA3 polyclonal antibodies for both methods were shown. It was found that when using the GRA3 polyclonal antibody, the OD value was still higher than that of the negative control by a factor of 2.1 at a dilution of 1:64,000. There was no significant difference between negative serum and PBS groups overall. It clearly indicated that the potency of the GRA3 polyclonal antibody is above 1:64,000. According to the ELISA results, we can see the optimal proportion of positive serum diluted to 1:8000 (Figure 3A,B).

### 3.4. Antibody Specificity Assay 

**Figure 4:**
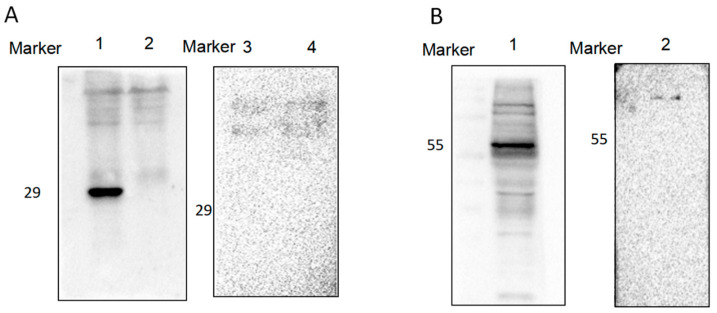
Specificity of the GRA3 PcAb evaluated by Western blotting. (**A**) The GRA3 PcAb reactivity against GRA3 proteins in N2a cells infecting the *T. gondii* ME49 strain. Lane M: protein marker; Lane 1: GRA3 protein (N2a cells infected with ME49 strain); Lane 2: no protein (normal cells without infection). Lane 3 and Lane 4 reflected that the negative serum served as the control. (**B**) The positive serum reacts with the total protein in N2a cells transfected with GRA3-GFP. The negative serum served as the control. Lane M: protein marker; Lane 1: GRA3-GFP recombinant protein (N2a cells transfected with GRA3-GFP plasmid); Lane 2 reflects that the negative serum served as the control.

The total protein of N2a cells infected with ME49 was identified by Western blotting using the GRA3 antibody and negative serum. The results showed that the GRA3 antibody specifically recognized the GRA3 protein with a molecular weight of about 29 kDa, which was in line with the expected size, while the negative sera did not recognize the target band at this position (Figure 4A). The result showed that the antibody can recognize the GRA3 protein expressed by ME49 parasites. The total protein of N2a cells after transfection with the GRA3-GFP plasmid was also identified by Western blotting using GRA3 PcAb compared with the negative serum. The results showed that the GRA3 antibody specifically recognized the eukaryotic expression of GRA3-GFP with a molecular weight of about 55 kDa, which was in line with the expected size, while the negative sera did not recognize the target band at this position (Figure 4B). The data indicated that the GRA3 PcAb could recognize the GRA3 protein expressed by ME49 parasites and eukaryotic cells.

### 3.5. Application in Indirect Immunofluorescence Assay

**Figure 5:**
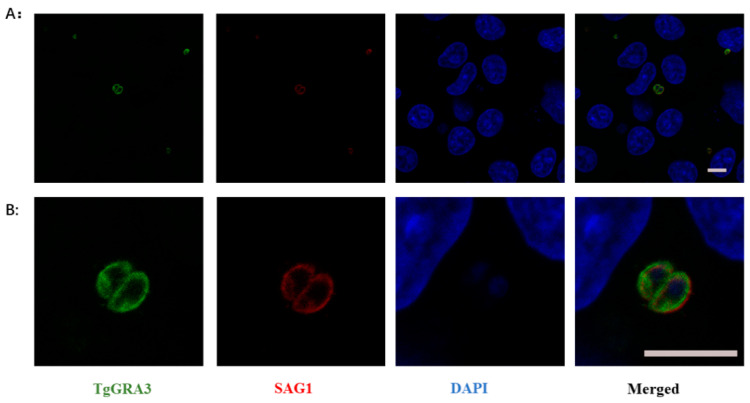
Confocal laser scanning microscopy of the *T.gondii* antigen recognized by GRA3 PcAb. Vero cells were infected with *T. gondii* ME49 strain tachyzoite for 24 h and parasites were detected by GRA3 PcAb. *T. gondii* was triple-stained with the GRA3 PcAb (green), SAG1 (red), and DAPI (blue). Scale bar = 10 µm. Group (**A**) is the full view. Group (**B**) is corresponding partial enlargement.

Twenty-four hours after the *T. gondii* invasion of the Vero cells, immunofluorescence assays were performed with the prepared anti-GRA3 PcAb and negative sera, respectively. The nuclei of the Vero cells and *T. gondii* parasites were blue under confocal microscopy (×600), the site of the SAG1 protein expression of the parasites was red, and the GRA3 antibody binding site was green (Figure 5). The result showed that GRA3 was localized to the periphery of the parasites, which is consistent with the previous reports of GRAs [20,21,22]. 

### 3.6. Immunoprecipitation Assay 

**Figure 6:**
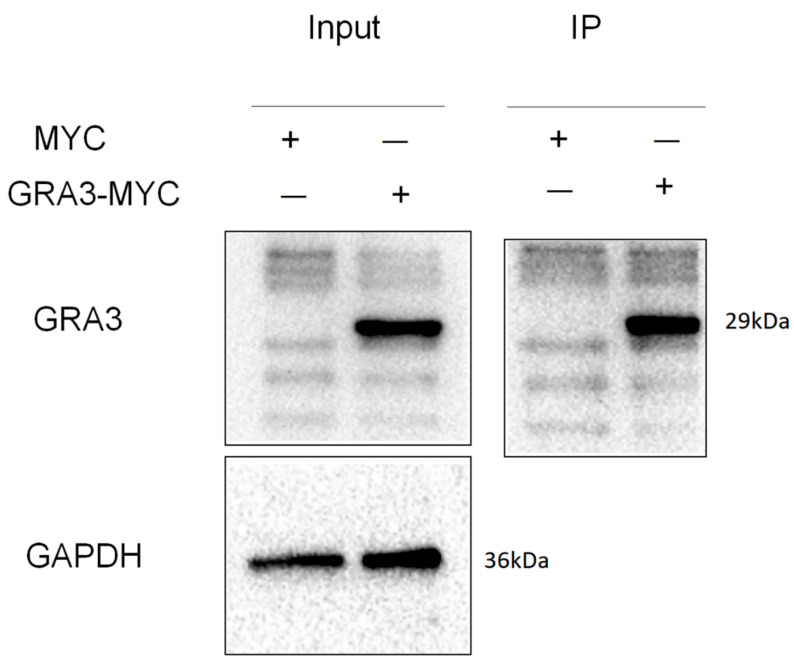
Immunoprecipitation assay of total protein from 293T cells infected by *T. gondi* ME49 strain using GRA3 PcAb. Input group contains GRA3 protein. IP group indicates that the precipitate contains this protein. GAPDH was used as a loading control.

The immunoprecipitation assay revealed that the GRA3 PcAb definitely interacts with the GRA3 protein in 293T cells transfected with MYC and GRA3-MYC plasmids (Figure 6). This result indicates that the antibody can be applied to immunoprecipitation.

## 4. Discussion

*T. gondii* can be divided into strong, weak, and non-virulent strains. Type II strains are lower in virulence than the others and require more parasites to reach lethal doses during severe acute infection in mice [23]. Notably, the genetic difference between type II and type I strains was only 1% [24]. *T. gondii* enter the cell by actively infiltrating the cytoplasmic membrane or phagocytosis by host cells, then change shape to become oval and are surrounded by the parasite vacuolar membrane (PV) from the host cells, thereby protecting them from host immunity [25]. The maturation of the vesicles is followed by the secretion of dense granules into the vesicle space and the resulting formation of a reticular structure within the vesicle space [26,27]. The accumulation of dense granule proteins in the expanding vesicles clearly indicates that granules continue to be released as the parasite replicates and the vesicles continue to expand [28,29,30,31].

An important component of the cyst wall is the dense granule protein of *T. gondii*. Variations in the relative composition of the dense granule proteins and protein structures of different strains of *T. gondii* have not been studied. Some dense granule proteins play a major function in cyst biosynthesis, but these proteins may be more abundant in strains that form cysts rapidly [32]. More than 50 GRAs have been identified, including GRA1-17, GRA39, GRA23-25, nucleoside triphosphate hydrolase (NTPase), cathepsins (CPC), STAT1 transcriptional activity inhibitor (IST), proteinase inhibitors (PI), Myc regulation (MYR), etc. GRA3 is a type I transmembrane protein encoding 29 kilodaltons located on the X chromosome and is a dense granule protein that is localized to the PVM and intra-vesicular network. As with other GRA proteins, it may acquire nutrients from the host or host organelle consortia on the PVM [22]. A previous study found that the GRA3 expression was significantly higher in strain Wh6 compared with the Chinese type 1 strain Wh3, suggesting that GRA3 is involved in the virulence of the strain in mice [33]. The bioinformatics analysis can be used to make a preliminary determination and prediction of the nature of GRA3. In particular, hydrophobicity analysis and transmembrane region prediction can predict whether the gene is a membrane protein, which is important for determining the direction of experimental research and the subsequent protein expression. The GRA3 gene was synthesized by biological information analysis, and a prokaryotic expression vector was constructed for the expression and purification of GRA3 protein and the preparation of multiclonal antibodies. The GRA3 protein was successfully expressed and purified by SDS-PAGE and Western blotting. Since GRA3-specific antibodies are temporarily unavailable on the market, and we also found that the protein expression of recombinant plasmids is difficult for meeting the requirements for antibody preparation in our preliminary experiments, we directly prepared peptides for the immunization of New Zealand rabbits to obtain GRA3 polyclonal antibodies. In order to obtain monoclonal antibodies specifically against the GRA3 protein, rabbits were immunized with the peptides and the immunized serum was used to amplify the GRA3 gene of the ME49 strain and construct an expression vector. The monoclonal antibody was measured by this indirect ELISA method. The purified protein was used as the antigen for encapsulation, and the potency of polycron antibodies obtained by indirect ELISA assay was up to 1:64,000. Western blotting and immunofluorescence tests showed that the obtained polyclonal antibodies had good specificity.

Therefore, in this study, according to the software analysis, the protein is generally hydrophilic and has two transmembrane structural domains. The expression was constructed after truncating the signal peptide and transmembrane region, and the antigenic peptide was synthesized and used to prepare various antibodies to obtain GRA3 polyclonal antibodies with a potency greater than 1:64,000. The western blotting validation results found that the specific band was detected at 29 kDa, consistent with the expected size. GRA3 was detected by immunofluorescence assay to be accurately localized to the periphery of the parasites in the infected cells. In recent years, researchers have done a lot of research on various proteins of *T. gondii*, and have reported the functions and properties of several *T. gondii* proteins, enabling us to comprehensively understand the pathogenic mechanisms of *T. gondii* at the protein level. Therefore, there is an urgent need for more in-depth studies on the exploration of *T. gondii* protein components and functions, immunological properties, and identification and evaluation of new antigens. *T. gondii* relies on different secreted proteins to assist itself throughout its growth cycle. *T. gondii* not only participates in the host’s immune response but also plays an important role in the growth and immune evasion process during the invasion of host cells. *T. gondii* secretory proteins are differentially expressed and function within different stages of *T. gondii* growth. Therefore, screening key proteins expressed in multiple stages of *T. gondii* with a high pathogenicity and immunogenicity as candidate antigens, as well as studying the interactions between proteins to develop antibodies to multiple antigens, are important ideas for future *T. gondii* antibody development. With the development of research, the GRAs family members were revealed one by one, and many novel proteins were successfully identified, such as GRA39, GRA35, GRA42, GRA41, etc. However, the functions and roles of these novel secretory proteins in the mechanisms of migration, invasion, replication, and immune evasion during the developmental stages of *T. gondii* are still unknown and deserve to be explored, which are valuable for understanding the pathogenesis, antigenic properties, induction of host immune response, and immune protection of *T. gondii.*

## 5. Conclusions

In this study, we have been able to develop a new polyclonal antibody against GRA3. This PcAb is suitable for Western blotting, immunofluorescence, and immunoprecipitation. Since the GRA3 protein plays a critical role in parasite growth and *T. gondii* infection, the preparation and preliminary application of the GRA3 PcAb may enhance our comprehension of the molecular mechanisms underlying *T. gondii* infections and clinical research on *Toxoplasmosis*.

## Data Availability

In this publication are all data supporting the result.

## Supplementary Materials

The following supporting information can be downloaded at: https://www.mdpi.com/article/10.3390/tropicalmed8030143/s1, Figure S1: Rabbit immunization protocol and blood collection procedure.
